# COVID-19 and Neurological Manifestations

**DOI:** 10.3390/brainsci13081137

**Published:** 2023-07-29

**Authors:** Kathleen Forero, Raghad Buqaileh, Clare Sunderman, Wissam AbouAlaiwi

**Affiliations:** Department of Pharmacology and Experimental Therapeutics, University of Toledo, Toledo, OH 43614, USA; kathleen.forero@rockets.utoledo.edu (K.F.); raghad.buqaileh@rockets.utoledo.edu (R.B.); clare.sunderman@rockets.utoledo.edu (C.S.)

**Keywords:** coronavirus, COVID-19, ACE2, neurology, cilia

## Abstract

Severe acute respiratory syndrome coronavirus 2 (SARS-CoV-2), a newly emerged coronavirus, has sparked a global pandemic with its airborne transmission and ability to infect with asymptomatic patients. The pathophysiology is thought to relate to the binding of angiotensin converting enzyme 2 (ACE2) receptors in the body. These receptors are widely expressed in various body organs such as the lungs, the heart, the gastrointestinal tract (GIT), and the brain. This article reviews the current knowledge on the symptoms of coronavirus disease 2019 (COVID-19), highlighting the neurological symptoms that are associated with COVID-19, and discussing the possible mechanisms for SARS-CoV-2 virus infection in the body.

## 1. Introduction

In December 2019, a novel coronavirus emerged in Wuhan, China, and has spread into a global pandemic. As of this writing, the World Health Organization estimated 767,750,853 confirmed cases around the world and 6,941,095 cumulative deaths [1]. The disease state, including its pneumonia-like symptoms, is called COVID-19, as it was a coronavirus discovered in 2019. The virus, SARS-CoV-2, is thought to be of zoonotic origin, originating from a bat-borne virus in China, to which it retains 88% genetic similarity [2]. The transmission of the virus is facilitated via direct contact or droplets released from sneezing or coughing, which is the rationale for the globally implemented social distancing mandates. The susceptibility of SARS-CoV-2 infection is directly proportional to age and the presence of various underlying pre-existing medical conditions, including asthma, diabetes, and hypertension.

**Figure 1:**
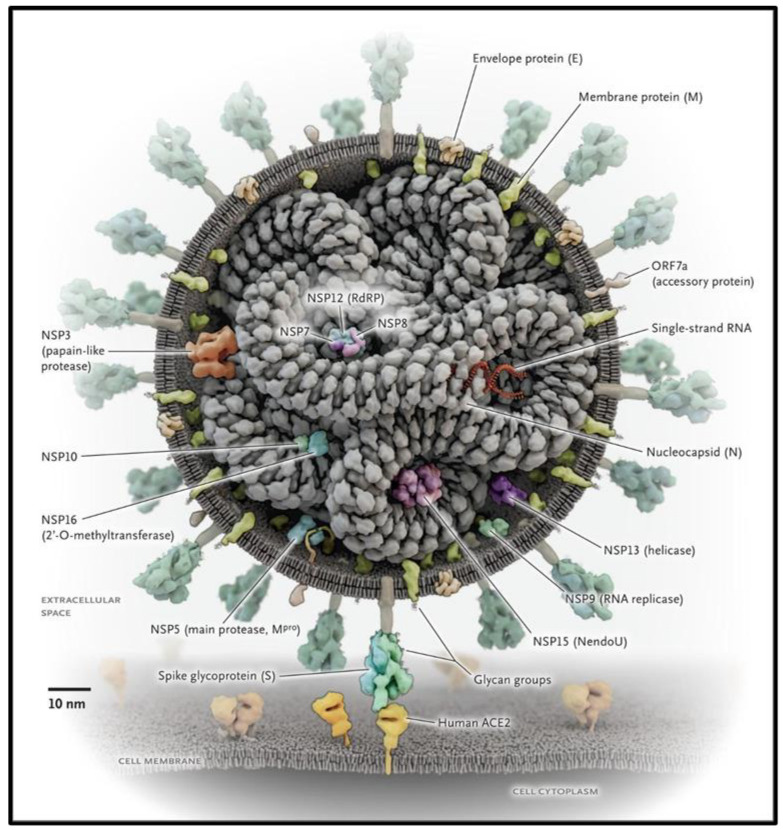
SARS-CoV-2 structure and proteins. Reprinted with permission from [5]. Copyright © 2020, Massachusetts Medical Society.

SARS-CoV-2 is one of seven known coronaviruses and is a member of the beta-coronavirus (βCoV) clade, which also includes Middle East respiratory syndrome coronavirus (MERS-CoV) and severe acute respiratory syndrome coronavirus (SARS-CoV) [2]. It appears to have similarities to other coronaviruses in symptoms and biological targets. SARS-CoV-2 is a virus with a single positive-stranded RNA genome that shares about 79.6% of its genome with SARS-CoV [3,4]. It is composed of four proteins, the spike (S), envelope (E), membrane (M), and nucleocapsid (N) proteins. The S protein is the structure that binds to the host cell receptor in order to enter and fuse with the host cell (Figure 1) [5]. Due to similarities in their genome, both SARS-CoV-2 and SARS-CoV bind to the ACE2 receptor, an extracellular enzyme on various organ tissues needed to enter the cell. 

The symptoms for SARS-CoV-2 infection range from fever and cough to anosmia and encephalopathy in severe cases. The flu symptoms are the predominant symptoms that physicians detect when diagnosing COVID-19 infections, but there are also neurological impacts of this virus that are as pertinent. Understanding the pathology of the SARS-CoV-2 virus throughout the body will improve the knowledge of its symptoms and improve efforts to develop a treatment. The goal of this article is to gather the current literature on the connection between SARS-CoV-2 infection and neurological manifestations to raise awareness for physicians on diagnosing these symptoms.

## 2. Cellular Pathways of Viral Infection into the CNS 

**Figure 2:**
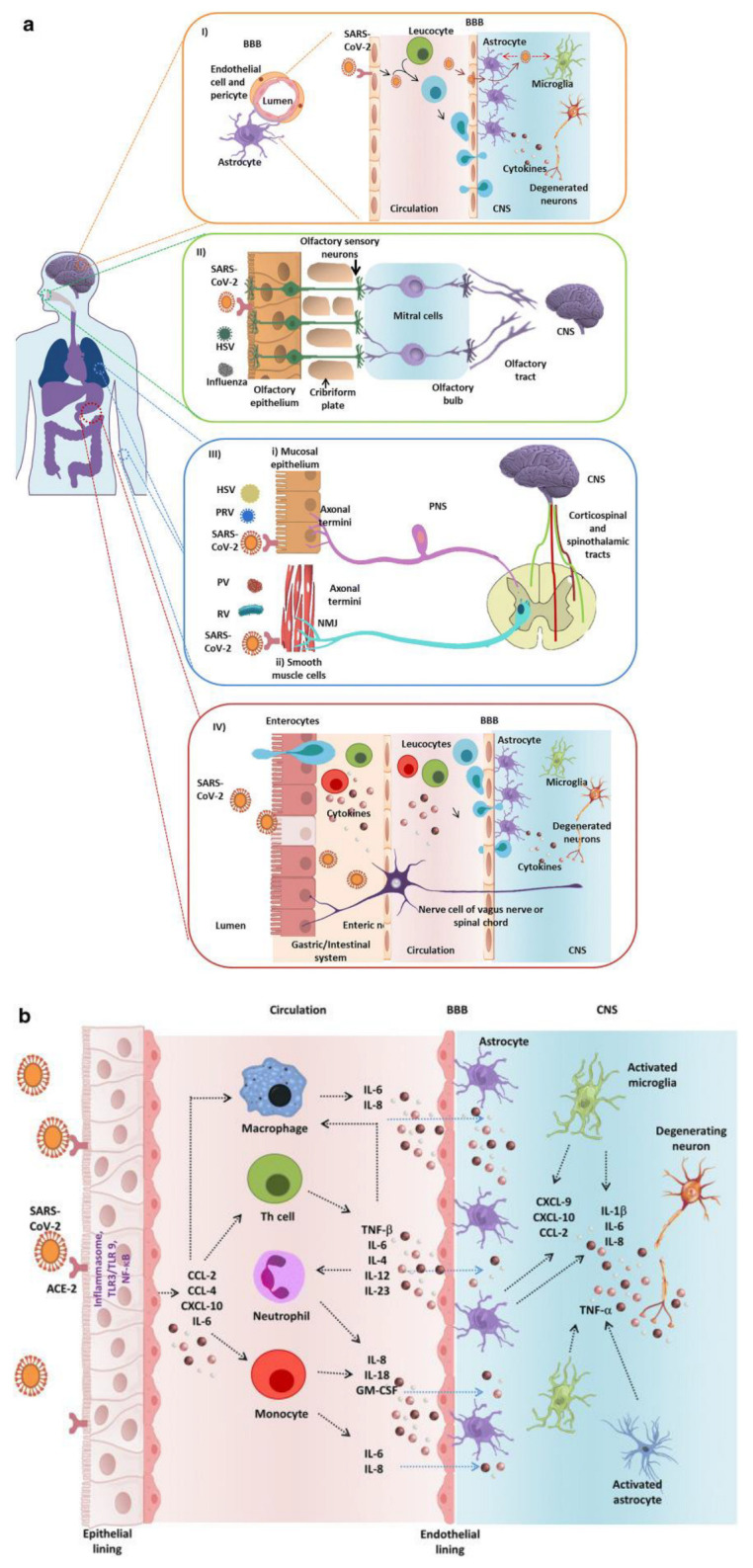
(**a**) SARS-CoV-2 entry pathways into the CNS. (**I**) Immune cells transport the virus through the BBB. (**II**) The virus can enter the CNS through the olfactory nerve terminals in the nasal cavity; (**III**) Viruses can enter the mucosal epithelium via the axonal termini of peripheral nerves and spread to the spinal cord by retrograde axonal transport. They can also infect smooth muscle cells and spread from muscles to PNS ganglia sensory/motor neurons via neuromuscular junctions (NMJ). (**IV**) the virus can infect cells in the gastrointestinal tract, reaching enteric neurons. (**b**) SARS-CoV-2-induced cytokine storm leading to inflammatory demyelination. Reprinted with permission from [9]. Copyright © 2020, Springer Nature Switzerland AG.

Studies have identified several routes for SARS-CoV-2 entry into the CNS. It is important to note that the viral infection may be due to one or a combination of these mechanisms. One pathway in which a virus can enter the CNS is through the olfactory nerve terminals in the nasal cavity, leading to viral RNA presence in the olfactory bulbs and brain regions linked to smell [6]. This pathway has reportedly facilitated the transport of xenobiotics, such as viruses or toxins, into the brain. Because the nasal cavity is composed of neuroepithelial cells that are also first-order neurons, the neurons directly connect to the brain, illustrating a vulnerability to certain agents to impact the brain [7]. Moreover, further studies on mice infected with SARS-CoV suggest that viral infection spread in the brain is primarily through olfactory bulb and leads to neuronal infections, which induces neuronal loss [8]. Additionally, SARS-CoV-2 can enter the systemic circulation, spreading throughout the body and through the blood–brain barrier (BBB). The virus may also infect immune cells such as monocytes and macrophages, which can then transport the virus to the BBB, facilitating CNS entry. Furthermore, the virus might propagate within the CNS through synapses, traveling along neuronal circuits from one region of the brain to another [7]. These methods are illustrated in Figure 2a.

SARS-CoV-2 neuro-invasion can impact the CNS by binding to the ACE2 receptor. This receptor is important in regulating blood pressure and is present throughout the body and in the capillary endothelium. Coronaviruses bind to ACE2, which can elevate blood pressure, including in the cerebrovasculature. This can increase the chance of a cerebral hemorrhage [6]. The SARS-CoV-2 virus also has S proteins that bind and damage the capillary endothelium, which can increase the permeability of the blood–brain barrier and allow the virus to move into the CNS [10].

Another explanation for the neurological manifestations reported in COVID-19 patients is that the virus does not need to enter the CNS, but these manifestations develop as a consequence of an indirect injury. As a result of the virus attacking the respiratory tract, it can cause hypoxia, or inadequate oxygen to other body tissues, which can have CNS effects, such as cerebral edema, intracranial hypertension, and acute ischemic stroke [6].

## 3. Symptoms and Neurological Manifestations among COVID-19 Patients

### 3.1. General Symptoms

SARS-CoV-2 is a type of coronavirus (CoV), which have been known for years—SARS, which is caused by SARS-CoV-1, appeared in 2003, and middle eastern respiratory syndrome (MERS), caused by MERS-CoV, appeared in 2012 [11,12]. The common manifestation of COVID-19 in patients are pneumonia-like symptoms, such as fever, cough, chills, and fatigue. Because it targets the lower respiratory tract, it can also lead to acute respiratory distress syndrome (ARDS) and dyspnea (or difficulty breathing) [2]. Lung injury caused by SARS-CoV-2 involves direct viral damage and inflammatory responses [13]. The virus attaches to ACE-2, which is expressed on the alveolar epithelium and vascular endothelium, and both are then taken into the cell. Similar to alveolar flooding in ARDS, cellular damage is caused by interstitial edema and alveolar fluid filling [13]. However, symptoms are not restricted to respiratory illnesses, there are reports of patients with gastrointestinal distress, kidney dysfunction, as well as neurological manifestations [2]. Records from previous pandemics and epidemics caused by viruses, including influenza (H1N1 and Spanish influenza), MERS-CoV, and SARS-CoV-1, have shown significant notable neuropsychiatric symptoms, such as anxiety, depression, insomnia, mania, psychosis, suicidality, and delirium, as well as central nervous system inflammatory disorders, such as encephalitis lethargica [12,14], narcolepsy, seizures, encephalopathy, and Guillain–Barre syndrome (GBS) [15]. One systematic review found that SARS-CoV-2 causes an indirect, proinflammatory effect that leads to intracranial, endothelial dysfunction and encephalopathy [16].

### 3.2. Statistics on Neurological Symptoms

Although there are limited data on the involvement of SARS-CoV-2 in the central nervous system neurological manifestations, evidence from new case reports and studies is showing the significant relationship between them, especially in patients with severe infection [15]. In a pooled study of 13,232 patients who had experienced long term COVID-19, 22% experienced cognitive impairment for at least 12 weeks [17]. In addition, many reports from Wuhan, China, have shown that approximately 20% of patients in a 799-patient retrospective study had alterations in their consciousness, meaning that they have shown encephalopathies symptoms, caused by cytokine storms [18]. Another study documented that 36.4% of 214 SARS-CoV-2 patients developed neurological symptoms such as headache, consciousness disorders, and paresthesia [6]. Additionally, there have been new reports pinpointing the symptoms of loss of smell (anosmia) and taste (dysgeusia) in COVID-19 patients [15]. These symptoms, ranging from headaches and dizziness to ischemic stroke and cerebral hemorrhage, impact both the central and peripheral nervous systems [19]. One case study reported a woman confirmed with SARS-CoV-2 with symmetric lesions in the thalami region of her brain shown through CT and MRI scans. This was due to a complication called acute necrotizing encephalopathy, which can occur from a viral infection, and leads to altered mental state and, thus, the breakdown of the BBB [20].

### 3.3. Anosmia

One of the most common otorhinolaryngological manifestations in COVID-19 patients is anosmia [12]. Anosmia is defined as the loss or inability to smell, and it is common in sinonasal diseases, some viral infections caused by some coronaviruses, parainfluenza, the Epstein–Barr virus, and many more [21], as well as in neurodegenerative diseases [22]. In a 417-patient study of patients of different ethnicities, 88% of COVID-19 patients reported olfactory dysfunction, and 80% of these patients had anosmia [23]. The mechanism by which SARS-CoV-2 adheres to the olfactory system leading to anosmia is still debatable [21,23], but many studies have shown viral infection’s ability to invade the central nervous system through receptor neurons in olfactory epithelium (Figure 2a) [24]. Human neuronal cells contain the angiotensin-converting enzyme 2 (ACE2) receptor, which is classified as a target and entry point for coronaviruses to the healthy cell, most importantly in SARS-CoV and SARS-CoV-2, at which the spike of the virus interacts with the receptor-binding domain, leading to their entry into the cell [6,8,10]. Another potential mechanism is inflammation in the olfactory epithelium [25]. Similar to the previous theory, high ACE2 receptor expression was reported in the olfactory epithelium (OE); cytokine release is caused by the virus binding to these cells, leading to a promotion in OE inflammation. Another theory about COVID-19-induced anosmia concerns the Nsp-13 protein and nasal cilia. The Nsp-13 protein damages the physiological interactions of the cilia structure by competing with endogenous-binding compounds of the centrosome proteins, leading to deciliation [25]. Anosmia as an olfactory impairment in SARS-CoV-2 patients might be the viral entry path to the CNS, leading to other neurological manifestations [12,14].

### 3.4. Cerebrovascular Manifestations

Cerebrovascular manifestations, such as strokes, are increasingly recognized in COVID-19 patients [10]. A cerebrovascular event refers to an acute compromise of the cerebral perfusion or vasculature, can be ischemic or hemorrhage, and is the fifth leading cause of death in the US [26]. In addition, a variety of CNS infections lead to strokes, such as the herpes virus [27]. It is not clear yet how SARS-CoV-2 infection leads to strokes, but epidemiological data and research evidence suggest that inflammation-triggering infections in the upper respiratory system, as with COVID-19, can lead to acute ischemic stroke, suggesting that this might be occurring because of the activation of thrombocytes as an immunological response to the infection alongside endothelial dysfunction [28]. This has been proven in the human influenza A (H1N1) virus that stimulates strokes as well; the inflammatory response was mostly the leading cause of strokes in H1N1 patients, through elevated cytokines levels [29]. One theory linking strokes to COVID-19 is that the virus induces acute ischemic strokes by promoting hypercoagulability [30]. Previous studies have found that some patients with severe COVID-19 had increased levels of pro-inflammatory cytokines, which can cause inflammation and hypercoagulability. Another theory is related to damaged endothelial cells being closely related to acute ischemic strokes. COVID-19 may damage brain endothelial cells by promoting cerebral thrombosis through inducing hypercoagulability [30].

### 3.5. Inflammatory Demyelinating Mechanisms

Myelin is crucial for the proper functioning of neurons as they encase the neuron and speed up the rate of electrical impulses. There is an increasing amount of evidence that contracting the SARS-CoV-2 viral infection includes a risk factor of demyelination in the central and peripheral nervous systems [31]. This is not surprising as demyelination has been seen in other coronavirus infections. The destruction of myelin, known as primary demyelination, is classified into various categories, but the most relevant category to COVID-19 is demyelination caused by inflammatory processes [32]. Inflammatory demyelination is usually caused by the immune system attacking the myelin sheath. The main mechanism behind this immune response occurs when T-lymphocytes stimulate an inflammatory cascade when they pierce the BBB, leading to demyelination [33]. The process of inflammatory demyelination looks very similar in COVID-19, as it does in multiple sclerosis. An early phase of COVID-19 is characterized by high-levels of pro-inflammatory cytokines in the blood, generating an overactive immune response [33]. The high levels of cytokines in COVID-19 are also referred to as a cytokine storm; characterized by a high expression of interlukin-6 and tumor necrosis factor α [34]. In normal conditions, the angiotensin-converting enzyme II (ACE2) receptors on the cell surface are occupied by angiotensin 2 (ANG II). However, in COVID-19, the ACE2 receptors are occupied by SARS-CoV-2. The levels of ANG II increase in the serum as a result of ACE2-mediated degradation [35]. SARS-CoV-2 activates NF-κB and STAT3 using pattern recognition receptors and the accumulated ANG II induces inflammatory cytokines. This is followed via the activation of the interluken-6 amplifier, which hyperactivates NF-κB and STAT3 (Figure 2b) [35].

## 4. Relationship between CoVs and Neurodegenerative Diseases

Due to the neuroinvasive nature of coronaviruses, it may lead to a degeneration of neurons that resemble different neurodegenerative diseases, such as Alzheimer’s disease (AD) and Parkinson’s disease (PD). An infection of HCoV-OC43 in hippocampal cell culture has reportedly targeted neurons, which is an affected area in AD brains. There also was an activation of caspase-3 proteins, which are a trigger for apoptosis or programmed cell death [36]. When inoculating HCoV-OC43 in mice, the size of the hippocampus was reduced compared to wild type mice, seen in a drop in the density of the neuronal layer of the hippocampus. These mice reportedly had deficits in cognition, such as memory encoding and learning [36]. These results indicate that a viral infection is able to foster symptoms that may lead to the development of AD in the future, as seen in the impact of the hippocampus. Parkinsonian symptoms were also demonstrated, with about 30% of surviving mice from a HCoV-OC43 infection exhibiting locomotor disabilities [36]. An elucidating detail about the treatment of PD is that tremors can be ameliorated with administration of amantadine, an antiviral agent [37]. Because of the unknown pathology of PD, the exact mechanism of how amantadine works in improving tremors in these patients is not fully understood, and its potential antiviral properties are not typically the primary reason for its effectiveness in managing Parkinson’s symptoms. It is believed to involve its effects on dopamine and acetylcholine receptors and glutamate activity. Amantadine’s use in Parkinson’s disease is primarily attributed to its effects on the brain’s neurotransmitters, since it increases the release and inhibits the reuptake of dopamine as well as reducing the activity of acetylcholine and blocking glutamate action to alleviate motor symptoms [38,39]. Additionally, as research progresses, our understanding of the connection of the antiviral mechanisms of action in PD may evolve. Early treatment with fluvoxamine, an SSRI used to treat depression, has also reportedly led to a decrease in death and intubation in COVID-19 patients [40]. 

## 5. Evaluation of ACE2 on SARS-CoV-2

The main theory on how SARS-CoV-2 enters cells and implements pathogenicity is by impairing the renin–angiotensin system (RAS). This system is important for maintaining a normal fluid and electrolyte balance, which is associated with the regulation of blood pressure [4].

### 5.1. The RAS System

The RAS system is an endocrine system initiated by the synthesis of angiotensinogen, a protein produced in the liver, which is released into the bloodstream. This peptide is cleaved by an aspartic protease produced in the kidney called renin, forming a decapeptide angiotensin I [41]. This inactive peptide is converted into angiotensin II by an angiotensin-converting enzyme (ACE), a dipeptidyl carboxypeptidase. ACE also acts by degrading bradykinin, which is a peptide that facilitates inflammation and vasodilation effects. When angiotensin II is formed, it will bind to type 1 angiotensin II receptor (AT1), which activates a signaling cascade that generates the release of aldosterone and causes vasoconstriction, thus elevating blood pressure [41]. Aldosterone is a steroid hormone secreted from the adrenal cortex that leads to the retention of sodium and water in the body, which ultimately increases blood pressure. However, this system also has a counterbalance system in place to negate these effects on the body. First, when the blood pressure has reached homeostasis levels, the kidney will lower its production of renin to slow down the effect. Another enzyme called ACE2 acts by cleaving angiotensin II into angiotensin (1–7) peptide, which has the opposite effect of angiotensin II. With the vasodilator effect of angiotensin (1–7) and the increased concentration of bradykinin, due to a drop in the angiotensin II degradation activity, the effect of ACE is reversed, and the blood vessels experience a vasodilation effect [42].

### 5.2. The Effect of SARS-CoV-2 on the RAS

ACE2, as stated earlier, is a receptor that binds to SARS-CoV-2 to allow for its entry into the host cell. These receptors are expressed in many organs throughout the body, such as the epithelial cells of the gastrointestinal tract (GIT) and vascular endothelial cells [43]. There are also ACE2 receptor expression in type II alveolar cells in the lungs, myocardial cells, epithelial cells in the esophagus, and proximal tubular cells in the kidneys [44]. The wide expression of these receptors may be a rationale for the varied symptoms reported among COVID-19 patients, from pneumonia-like symptoms to GIT issues and neurological effects. The effect of ACE2 on the pathogenesis of this disease is complex. Populations with high expression levels of ACE2, such as men and people of Asian ethnicity, are more susceptible to infections because the virus binds to ACE2 receptors to enter the cells and replicate. However, it is also reported that the ACE2 presence has a protective role against acute lung injury, indicating that its relationship with the pathology of COVID-19 is ambiguous and complicates the effort to discover a cure [4].

The RAS system has implications in the brain as well, which can improve the understanding of the neurological symptoms from SARS-CoV-2 infections. ACE2 receptors are expressed in the brain and in the cytoplasm of neuronal cells as well as in astroglial cells, with the highest neuronal activity levels in the hippocampus [45]. Components of the RAS are also present in the brain, such as angiotensin (1–7) and renin. Angiotensin (1–7) is produced in the blood vessels in the brain, in areas such as the hypothalamus, amygdala and the medulla oblongata, and has vasodilatory effects. It enhances the bradykinin activity of vasodilation by inhibiting ACE, promotes the release of nitric oxide and activates the release of prostaglandins [45]. ACE2 receptors were also reported to impact cardiovascular controlling brain regions, such as the nucleus tractus solitarius, which illustrates the brain RAS effect on the maintenance of blood pressure [46]. Overall, this demonstrates that the RAS has both endocrine/cardiovascular and neurological effects, which may be impacted by infection with SARS-CoV-2.

### 5.3. Connection to Ciliated Cells

**Figure 3:**
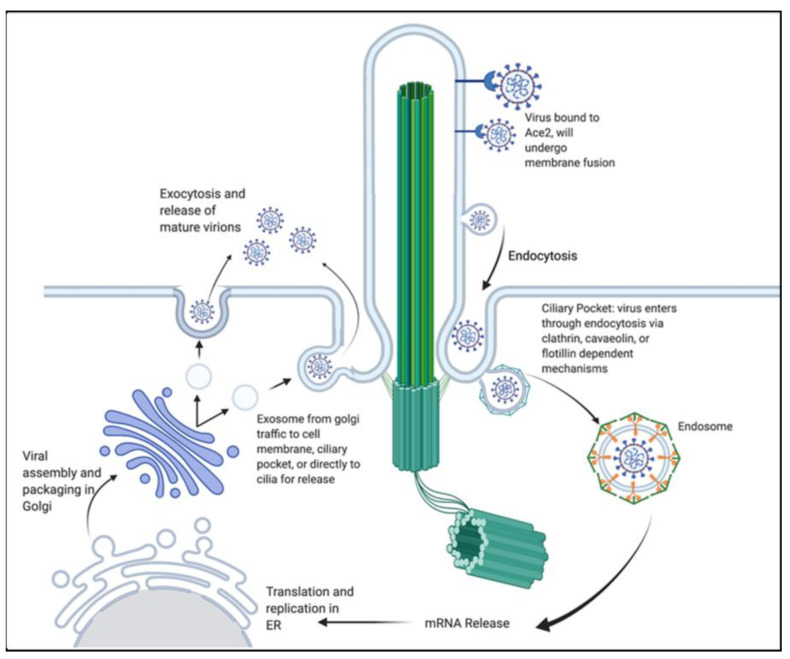
Proposed coronavirus entry into cells through primary cilia pathway. Detailing entry through ciliary membrane fusion or endocytosis in the ciliary pocket. As well as exocytosis of the mature virions through ciliary mechanisms. Reprinted with permission from [56]. Copyright © 2021, The American Physiological Society.

There is an interesting connection between ciliated cells and the RAS, as well as with coronaviruses, which needs to be expanded upon. A study investigating the effect of a human coronavirus (HCoV 229E) inoculation on ciliated nasal epithelium cells reported that the viral infection resulted in a disruption in the structure of the epithelial cells in the nasal cavity as well as the activity of the intact cilia. The ciliary structure was impaired, with an increase in ciliary dyskinesia reported in the infected individuals but not in the control group [47]. These ciliated nasal epithelial cells also express a high level of ACE2 receptors, which highlights its susceptibility to SARS-CoV-2 infection [48]. With SARS-CoV infection, whose mechanism may hold similarities to SARS-CoV-2, was reported to localize its structural proteins on the ciliated epithelial cells [49]. A recent study reported the consistent localization of ACE2 in ciliated epithelial cells of the respiratory system, in pulmonary motile cilia, and in the primary cilia of kidney IMCD3 cells [50]. Mature virions have also been reported to be “trapped” among the cilia [51,52,53], all of which supports the idea that cilia could be a first point of viral attachment. Due to the internal structure of primary cilia, they typically sit in a depressed or concaved area of the cell membrane; this area is known as the ciliary pocket and functions as an endocytic domain. When SARS-CoV-2 is cleaved by other proteases than TMPRSS2, the entry pathway is redirected to an endocytic mechanism that can be mediated by clathrin. The cilia pocket is enriched with clathrin-coated pits, making it highly significant for both endo and exocytosis [54,55], thus making cilia a potential two-way street for both viral entry and exit from the cell (Figure 3). These findings draw a connection between cilia in epithelial cells in the respiratory system to the viral spread of SARS-CoV-2. Understanding the mechanism of pathology of SARS-CoV-2 to these ciliated cells can propose the usage of cilia as a target for treating this viral infection.

## 6. Conclusions

SARS-CoV-2 viral infection generated a global panic and a scientific push to develop a cure. Defining the mechanism of the pathogenicity of SARS-CoV-2 would improve the ability of physicians to treat patients and of researchers to develop a drug to target the disease state. Building on the current literature on the topic can help determine a viable drug target for SARS-CoV-2, whether that be ACE2 receptors or ciliated epithelial cells.

## Data Availability

No new data were created or analyzed in this study. Data sharing is not applicable to this article.
